# Three-dimensional flow velocity determination using laser-induced fluorescence method with asymmetric optical vortex beams

**DOI:** 10.1038/s41598-024-52179-0

**Published:** 2024-01-23

**Authors:** Kenichiro Terasaka, Shinji Yoshimura, Hiroki Minagawa, Mitsutoshi Aramaki

**Affiliations:** 1 Interdisciplinary Graduate School of Engineering Sciences, Kushu University, Kasuga, Fukuoka 816-8580 Japan; 2 National Institute for Fusion Science, Toki, Gifu 509-5292 Japan; 3 Center for Low-temperature Plasma Sciences, Nagoya University, Nagoya, Aichi 464-8601 Japan; 4 College of Industrial Technology, Nihon University, Narashino, Chiba 275-8575 Japan; 5 Present Address: Department of Computer and Information Sciences, Sojo University, Kumamoto, Kumamoto 860-0082 Japan

**Keywords:** Fluorescence spectroscopy, Plasma physics

## Abstract

Laser-induced fluorescence (LIF) Doppler spectroscopy using an optical vortex beam with an asymmetric intensity distribution, referred to as aOVLIF, is proposed as a new method to measure plasma flow velocity. LIF spectra were calculated numerically using typical laboratory low-temperature plasma parameters, and it was revealed that an ion flow across the beam produces a frequency shift of the spectra. This method also has the capability of temperature measurements. The propagation effects of asymmetric optical vortex beams are discussed assuming an actual experiment, and it is found that the sensitivity to the transverse flow velocity is approximately unchanged. The aOVLIF method, which exploits the inhomogeneous phase structure of optical vortices, can be applied to the determination of three-dimensional velocity vectors and promises to enhance the usefulness of conventional LIF spectroscopy using plane waves.

## Introduction

Plasma, known as *the fourth state of matter*, is an ionized gas consisting of electrons, ions, and neutral particles. Flow is an essential aspect of plasmas in typical applications, including semiconductor manufacturing processes^1^, electric propulsion^2,3^, and magnetic confinement fusion research^4^. A variety of electrical and optical methods have been proposed to measure plasma flow accurately^5,6^.

Laser-induced fluorescence (LIF) Doppler spectroscopy using a narrowband tunable laser is a nonintrusive and unperturbed diagnostic tool to accurately measure the flow velocity of ions and neutral particles and has become a standard method in plasma research, especially for low-temperature plasmas^7–16^. In the LIF method, the energy state of atoms is excited by a laser photon whose energy is equal to the difference between two atomic energy levels, and the fluorescence emitted by the de-excitation process is then observed^17,18^. By sweeping the laser frequency, the number density distribution of atoms satisfying the resonant absorption condition is visualized as an LIF spectrum^10^. The frequency shift and the width of the LIF spectrum determine the flow velocity and the temperature, respectively.

In conventional studies, the LIF spectra are interpreted based on the phase structure of plane waves. The Doppler shift of the resonant absorption frequency for a beam with phase structure $e^{\text {i}\psi }$ is given by $\delta = \nabla \psi \cdot \varvec{\upsilon }$^6^. Here, $\psi$ is the phase factor, $\varvec{\upsilon }$ the velocity of the atom, and $\delta$ the frequency shift in rad/s. Since the Doppler shift is $\delta = \varvec{k} \cdot \varvec{\upsilon }$ for a plane wave with wavenumber $\varvec{k}$, conventional LIF is, in principle, a one-dimensional measurement in the direction of beam propagation. Thus, only the velocity component projected onto the optical axis can be obtained with this method, and flow perpendicular to $\varvec{k}$ never be detected. Therefore, multiple optical paths are needed to determine the three-dimensional flow vector. However, the configuration of the equipment often limits the laser path. If an LIF method with sensitivity to the transverse flow is established, it expands the versatility of the method.

An optical vortex (OV), which carries orbital angular momentum, is a propagation mode with a helical phase structure and doughnut-shaped intensity distribution^19^. Allen et al. determined that an atom moving in an OV beam experiences additional Doppler effects due to phase inhomogeneity^20^. For a Laguerre-Gaussian beam with topological charge (TC) $\ell$ propagating in the *z* direction, the phase gradient at the beam waist is given by $\nabla \psi \approx k \varvec{e}_z + (\ell / r) \varvec{e}_\varphi$ in cylindrical coordinates $(r, \varphi , z)$. Hence, the resonant absorption frequency of a moving particle is modified by an additional Doppler shift of $\ell \upsilon _\varphi / r$, which depends on the particle position in the beam cross-section and the transverse flow velocity.

In absorption spectroscopy, it has been reported that the spatial dependence of the resonance absorption condition for an OV beam can be straightforwardly utilized to measure the neutral gas flow across the beam in a plasma^21^. Absorption spectroscopy is applicable when sufficient absorption is ensured along a single optical path. On the other hand, LIF spectroscopy is a practical option when sufficient absorption is unavailable. In typical experiments, the LIF photons from the entire beam cross-section are observed by the collecting optics. Yoshimura et al., proposed an LIF method using optical vortex beams (OVLIF), where the transverse flow speed is determined from the broadening of the LIF spectrum^22^. However, the OVLIF loses information on the flow direction and has not been able to achieve three-dimensional flow determination.

In this paper, we propose an advanced OVLIF using asymmetric optical vortex (aOV) beams referred to as asymmetric OVLIF (aOVLIF), which limits the LIF emission region and utilizes the spatial dependence of the resonant absorption condition. The LIF spectra are numerically calculated using the parameters in actual experiments. The results show that the aOVLIF method enables us to obtain the flow velocity across the beam from the center frequency shift of the LIF spectrum rather than its broadening. Moreover, it is demonstrated that the aOVLIF method has the potential to determine the three-dimensional velocity vector despite using a single optical path. It is also revealed that the characteristics of the LIF spectrum do not depend on the distance from the beam waist.

**Figure 1:**
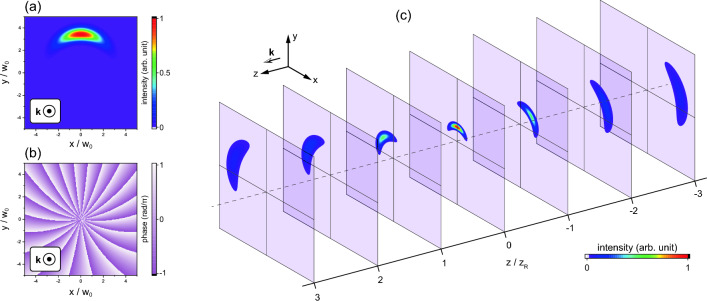
2D distributions of (**a**) the intensity and (**b**) the phase for an $\ell =20$ aOV beam at $z=0$. The asymmetry parameter is $[\text {Im}(x_\text {s}), \text {Im}(y_\text {s})]=[-w_0/2, 0]$. The propagation of the beam is depicted in (**c**).

**Figure 2:**
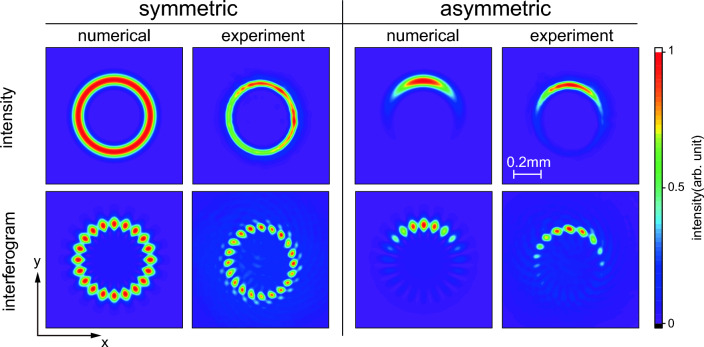
(upper) 2D intensity distribution of numerically calculated and experimentally observed symmetric and asymmetric OVs ($\ell =20$). (lower) The interferograms obtained by superposing a Gaussian reference beam and an OV beam. The asymmetry parameter for the calculations is $[\text {Im}(x_\text {s}), \text {Im}(y_\text {s})]=[-w_0/5, 0]$.

**Figure 3:**
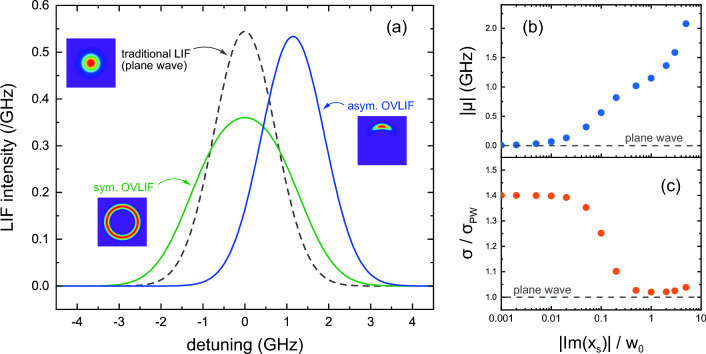
(**a**) Comparison of numerically calculated spectra in traditional LIF with Gaussian beam (gray, dashed), OVLIF with symmetric LG beam (green, solid), and aOVLIF methods (blue, solid). $V_x=5$ km/s, $V_y=V_z=0$, $T=0.1$ eV, and $w_0=5$ $\mu$m. The TC is $\ell =20$, and the shift parameter is set to $[\text {Im}(x_\text {s}), \text {Im}(y_\text {s})]=[-w_0/2, 0]$. For aOVLIF, the asymmetry parameter dependences of the frequency shift ($\mu$) and width ($\sigma$) of the spectrum are shown in (**b**) and (**c**), respectively. The width is normalized by that of the traditional LIF spectrum ($\sigma _\text {PW}=0.73$ GHz).

## Results

### Concepts of aOVLIF method

The aOVs known to be stable free-space propagation modes have an azimuthally inhomogeneous intensity distribution. In this paper, we consider asymmetric Laguerre–Gaussian modes (or shifted Laguerre–Gaussian modes)^23–25^ as aOV beams. The complex amplitude of an aOV beam with wavenumber *k* propagating in the *z* direction is given by

1 $$\begin{aligned} u(r, \varphi , z)&= \frac{w_0}{w} \left( \frac{\sqrt{2}}{w} \right) ^{|\ell |} \left[ (x-x_\text {s}) + \text {i} \sigma _\ell (y-y_\text {s}) \right] ^{|\ell |} L_p^{|\ell |} \left( \frac{2\rho ^2}{w^2} \right) \exp \left( -\frac{\rho ^2}{w^2} + \frac{\text {i}k\rho ^2}{2R} - \text {i} \theta _\text {G} \right) , \end{aligned}$$

2 $$\begin{aligned} \rho ^2&= (x-x_\text {s})^2 + (y-y_\text {s})^2, \end{aligned}$$

3 $$\begin{aligned} w&= w_0 \sqrt{1+\hat{z}^2}, \end{aligned}$$

4 $$\begin{aligned} R&= z \left( 1 + \frac{1}{\hat{z}^2} \right) , \end{aligned}$$

5 $$\begin{aligned} \theta _\text {G}&= (2p+|\ell |+1) \tan ^{-1} (\hat{z}), \end{aligned}$$

where $\hat{z}=z/z_\text {R}$ ($z_\text {R}=kw_0^2/2$; Rayleigh range) is the normalized axial position^24^. The coordinates of the system are shown in Fig. 1. The origin in the axial direction ($z=0$) is located at the beam waist. $w_0$ and $\sigma _\ell$ stand for the Gaussian beam waist and the sign of the TC, respectively. The function $L_p^{|\ell |}$ is the associated Laguerre polynomial giving the radial mode *p* and the TC, and we consider aOV beams with $p = 0$ in this paper.

The asymmetry of the intensity distribution is characterized by the imaginary part of the parameter $(x_\text {s},y_\text {s})$. Here, we introduce the asymmetry parameter $D_\text {s}$ defined by $D_\text {s}=\sqrt{[\text {Im}(x_\text {s} )]^2+[\text {Im}(y_\text {s} )]^2 }$. In this paper, we adopt the relationship $x_\text {s}=-\text {i} \sigma _\ell y_\text {s}$, and this assumption allows that the minimum intensity position to be $(x, y)=(0, 0)$ except at infinity. Note that the results presented in this paper do not depend on the choice of origin. Figure 1a,b show the intensity and phase distributions of an asymmetric Laguerre–Gaussian mode for $\ell =20$ and $D_\text {s}=w_0/2$ at the beam waist, respectively. Although the intensity is localized in the azimuthal direction, the phase gradient is preserved; the TC of the beam in Eq. (1) is $\ell$^24^. As shown in Fig. 1c, the aOV beam rotates during propagation, similar to the propagation of symmetric OV beams with defects^26^. The effect of such propagation characteristics on the LIF spectrum is a subject for consideration.

We produced OV beams in the laboratory (see “Methods”) and examined the achievable values of the parameters $w_0$, $\ell$, and $D_\text {s}$. Figure 2 shows the experimentally and numerically obtained intensity distributions and interference patterns at the beam waist for $\ell =20$ OV beams, in which the interference pattern provides the information about the phase structure. A comparison between the experimental and numerical results shows that an aOV beam is successfully generated and that the ranges $\ell \le 50$ and $D_\text {s} < w_0$ are appropriate for examination.

To demonstrate the fundamental concepts of the aOVLIF method effectively, we validated LIF spectra with realistic beam parameters in a practical experiment. Figure 3a shows LIF spectra obtained with Gaussian, symmetric OV, and aOV beams. The Gaussian beam waist and the ion flow velocity are set to $w_0=50$ $\upmu$m and $V_x=-5$ km/s. The TC and asymmetry parameter are $[\ell , \text {Im}(x_\text {s}), \text {Im}(y_\text {s})] = [20, -w_0/2, 0]$ in aOVLIF and [20, 0, 0] in OVLIF. Because the parallel flow velocity is set to $V_z = 0$, there is no frequency shift in the LIF spectrum for the Gaussian beam. It is clear that the presence of transverse flow produces a frequency shift in the spectrum in aOVLIF, whereas it is reflected in a broadening of the spectrum in OVLIF.

To investigate the characteristics of the LIF spectra, we calculated the frequency moments of the spectra defined below:

6 $$\begin{aligned} \mu&= \int _{-\infty }^\infty \nu I_\text {LIF} (\nu ) \text {d}\nu , \ \ \sigma ^2 = \int _{-\infty }^\infty (\nu -\mu )^2 I_\text {LIF} (\nu ) \text {d}\nu , \nonumber \\ S&= \frac{1}{\sigma ^3} \int _{-\infty }^\infty (\nu -\mu )^3 I_\text {LIF} (\nu ) \text {d}\nu , \ \ K = -3 + \frac{1}{\sigma ^4} \int _{-\infty }^\infty (\nu -\mu )^4 I_\text {LIF} (\nu ) \text {d}\nu , \end{aligned}$$

where $I_\text {LIF}(\nu )$ is the LIF spectrum satisfying $\int _{-\infty }^\infty I_\text {LIF} (\nu )\text {d}\nu = 1$. The laser frequency $\nu$ is detuned by the absorption frequency for the stationary ions. For the numerically obtained LIF spectra, the statistical quantities are calculated using the discrete expressions in Eq. (6) (see “Methods”). Figure 3b,c show the asymmetry parameter dependence of the shift ($\mu$) and width ($\sigma$) of the aOVLIF spectrum, respectively. The traditional LIF spectrum takes $S=K=0$ since a Maxwell–Boltzmann distribution is assumed as the velocity distribution function (see “Methods”). As the beam asymmetry increases, the frequency shift of the spectrum becomes large and the width of the spectrum becomes close to that in a traditional LIF.

The spectrum obtained using an OV beam is generally broader than that obtained using a Gaussian beam due to the non-uniform phase distribution in the former case [see Eq. (16)]. However, this broadening is negligible for $w_0=50$ $\upmu$m (minimum value in our laboratory), as will be shown later. In other words, this result indicates that thermal broadening of the spectrum blurs the effect of transverse flow in conventional OVLIF. Evaluating the flow based on the frequency shift improves the sensitivity regarding the transverse flow velocity in the OVLIF method. It is also confirmed that in the aOVLIF method, the skewness *S* and kurtosis *K* are almost zero and that the LIF spectrum coincides with the assumed velocity distribution function (the values of *S* and *K* for different plasma parameters are investigated in Fig. 7).

These facts imply that the aOVLIF method can simultaneously determine the flow velocity and temperature. Furthermore, the aOVLIF method is applicable to arbitrary distribution functions since the absorption condition is independent of the functional form of the velocity distribution function. It is noted that, in the present calculation parameter range, the phase gradient parallel to the wavenumber vector is dominant. For example, in the case of an anisotropic velocity distribution function where the temperature differs in parallel and perpendicular to the wavenumber vector, the width of the LIF spectrum reflects the temperature in the parallel direction.

### Transverse flow evaluation using aOVLIF method

**Figure 4:**
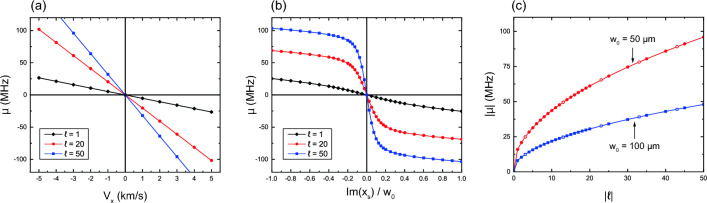
Frequency shift of the LIF spectrum ($\mu$) as a function of (**a**) transverse flow velocity, (**b**) asymmetry parameter, and (**c**) TC. The beam width and asymmetry parameter in (**a**) are $w_0=50$ $\mu$m and $[\text {Im}(x_\text {s}), \text {Im}(y_\text {s})]=[-w_0/2, 0]$. The diamonds (black), circles (red), and squares (blue) are for $\ell =1$, 10, and 50, respectively. In (**b**), the beam width is $w_0=50$ $\upmu$m, the transverse flow is $V_x=-3$ km/s ($V_y=V_z=0$), and the asymmetry parameter is $[\text {Im}(x_\text {s}), \text {Im}(y_\text {s})]=[-w_0/2, 0]$. In (**c**), the closed and open symbols are for $\ell >0$ and $\ell <0$, respectively, and the asymmetry parameter and transverse flow velocity are the same as in (**a**) and (**b**), respectively.

Figure 4 shows the additional Doppler shift of the LIF spectrum at the beam waist ($z=0$) as functions of (a) transverse flow velocity, (b) asymmetry parameter for $D_\text {s}=|\text {Im}(x_\text {s})|$, and (c) absolute value of TC. Figure 4a indicates that the frequency shift is proportional to the flow velocity and its sign changes with flow direction. As already shown in Fig. 3, the magnitude of the frequency shift increases as the asymmetry parameter increases, shown in Fig. 4b). For $|\text {Im}(x_\text {s})| > 0.2$, the frequency shift tends to saturate. It is experimentally desirable to consider this region because the ambiguity of the asymmetric parameter does not lead to significant errors in flow velocity determination. A smaller beam with a higher TC provides efficient frequency shifts, as shown in Fig. 4c, since such a beam has a larger phase gradient parallel to the transverse flow. The sign of the shift depends on both the asymmetry parameter and the TC for a given flow velocity.

To establish the aOVLIF method, it is necessary to quantitatively link the frequency shift of the LIF spectrum to the transverse flow velocity. To investigate this requirement, we introduce a dimensionless quantity:

7 $$\begin{aligned} Q= \frac{2\pi \mu w_0}{V_x}. \end{aligned}$$

**Figure 5:**
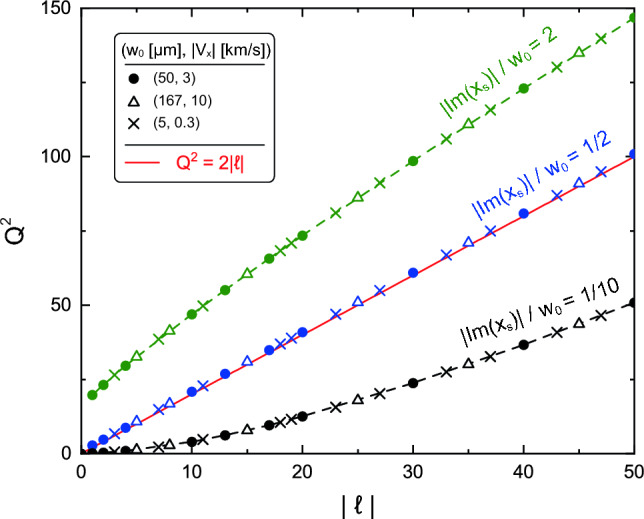
Quantity $Q^2=(2\pi \mu V_x/w_0 )^2$ as a function of magnitude of TC, $|\ell |$, for each value of asymmetry parameter. The transverse flow is in the *x* direction and the asymmetry parameter is set to $D_\text {s} = |\text {Im}(x_\text {s})|$. The solid red line indicates the relationship $Q^2=2|\ell |$. (It is not a fitting curve to the data for $|\text {Im}(x_\text {s})|=w_0/2$). The dashed lines are guides for the eye and show $|\text {Im}(x_\text {s})|=2w_0$ and $w_0/10$.

We plot $Q^2$ as a function of TC for each asymmetry parameter in Fig. 5. This result shows that if the three beam parameters $w_0$, $\ell$, and $D_\text {s}$ are experimentally known, the transverse flow velocity is uniquely determined from the frequency shift. It can also be seen that $Q^2$ increases with increasing magnitude of the asymmetry parameter. This property can be attributed to the fact that an aOV beam with a larger $D_\text {s}$ has a larger azimuthal phase gradient in the strong intensity region. When the asymmetry parameter is $|\text {Im}(x_\text {s})| = w_0/2$, the following relationship holds:

8 $$\begin{aligned} Q^2 \approx 2|\ell |. \end{aligned}$$

here, Eq. (8) is more accurate when $|\text {Im}(x_\text {s})|=0.47w_0$. Being able to express the frequency shift by a simple relationship will be helpful for experiments. In addition, the Doppler shift of the resonant absorption frequency at the maximum intensity position of propagating aOV beams also obeys the same form as Eq. (8), which will be discussed later.

**Figure 6:**
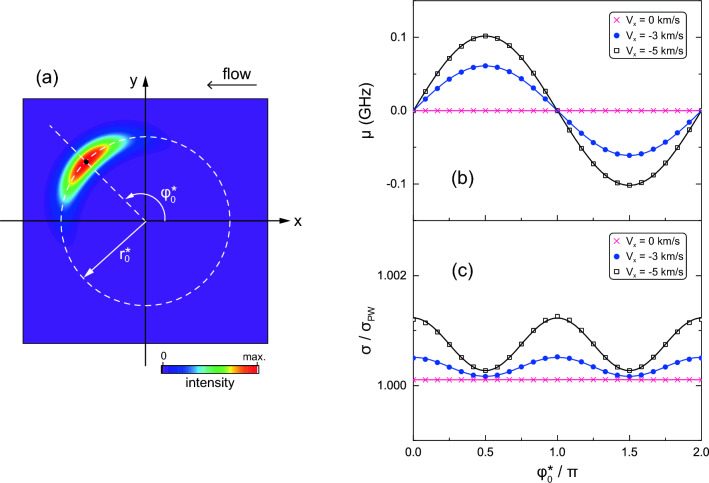
Frequency shift ($\mu$) and normalized width ($\sigma / \sigma _\text {PW}$) of LIF spectra as function of $\varphi ^*_0$ for aOV beam with of $w_0=50$ $\mu$m, $D_\text {s}=w_0/2$, $\ell =20$, and $z=0$. The definitions of $r^*_0$ and $\varphi ^*_0$ are shown in (**a**) and the frequency shift and width are shown in (**b**) and (**c**), respectively.

The discussion so far considered the case where $\text {Im}(y_\text {s})=0$ for the asymmetry parameters. On the other hand, the intensity distribution in the *x*-*y* plane can be chosen arbitrarily in the azimuthal direction by adjusting the asymmetry parameter. We define the beam position $(r^*_0, \varphi ^*_0)$ by the radius and azimuthal angle at which the intensity is a maximum, and a schematic of the coordinate is presented in Fig. 6a. The frequency shift and width of the LIF spectrum as functions of $\varphi ^*_0$ are shown in Fig. 6b,c, respectively, where the transverse flow velocity is in the *x* direction ($V_y = 0$).

It is emphasized that if the scale length of the plasma flow is sufficiently large compared to the beam size, the aOVLIF method can determine the three-dimensional velocity vector without using any other assumptions on the flow field. In the general case where the flow is three-dimensional ($V_x$, $V_y$, $V_z\ne 0$), the frequency shift of the LIF spectrum is given by $\mu (\varphi _0^*)=\mu _\perp \sin (\varphi _0^* - \varphi _\text {flow}) + \mu _\parallel$, where the parameters $\mu _\perp$, $\varphi _\text {flow}$, and $\mu _\parallel$ indicate the amplitude, phase shift, and offset, respectively. Hence, the frequency shifts due to the parallel and transverse flow velocities can be distinguished by using the relationships $\mu _\parallel = [\mu (\varphi _0^*)+\mu (\varphi _0^*+\pi )]/2$ and $\mu _\perp \sin (\varphi _0^* - \varphi _\text {flow})=[\mu (\varphi _0^*)-\mu (\varphi _0^*+\pi )]/2 \equiv \beta$, respectively. In experiments, four measurements at $\pi /2$ intervals for $\varphi _0^*$ are sufficient to determine the sinusoidal function of $\mu (\varphi _0^*)$ from the curve fitting. When the quantities $\beta _1=\beta (\varphi _0^*)$ and $\beta _2= \beta (\varphi _0^*+\pi /2)$ are obtained, the frequency shifts of the LIF spectrum due to the *x*-component and the *y*-component of transverse flow are evaluated by $\beta _1 \sin (\varphi _0^*)+\beta _2 \cos (\varphi _0^*)$ and $-\beta _1 \cos (\varphi _0^*) +\beta _2 \sin (\varphi _0^*)$, respectively. If only a specific component of the transverse flow is required, two measurements can provide sufficient information by aligning the phase gradient of the aOV beam parallel to that direction. In addition, an alternative method may also be useful to use a sign reversal of the topological charge (TC) by using a property of $\mu _\perp \sin (\varphi _0^*-\varphi _\text {flow})|_\ell = - \mu _\perp \sin (\varphi _0^*-\varphi _\text {flow})|_{-\ell }$. In addition, alignment errors of $\varphi _0^*$ can result in undesired frequency shifts of the LIF spectrum. For example, an aOV beam with a poorly adjusted phase gradient would affect the frequency shift of the LIF spectrum for large $V_y$, even at $\varphi _0^* = \pi /2$. The key to quantitatively determining the flow velocity is how accurately the phase gradient perpendicular to the wavenumber vector can be adjusted.

The sinusoidal dependence of the frequency shift is a general characteristic of the aOVLIF method. This fact ensures that the flow velocity vector across the beam can be determined. Moreover, when parallel flows exist simultaneously, this component is added as an offset to the sinusoidal curves in Fig. 6. Therefore, the aOVLIF method completely overcomes the problems of the conventional OVLIF method for transverse flow measurements and is also effective in evaluating three-dimensional flow vectors in a single optical path.

The width of the spectrum fluctuates with a period of $\pi$ in Fig. 6c, and the amplitude becomes large for a faster transverse flow velocity. This characteristic is due to the integration in the beam cross-section. Although broadening of the spectra is inevitable in aOVLIF, for some large values of asymmetric parameters (typically $D_\text {s} > w_0/5$), the broadening is not significant in the temperature evaluation. Given the accuracy of the actual experiments, the amplitude is negligibly small for typical low-temperature plasma. Therefore, it can be concluded that aOVLIF provides a way to measure the transverse flow velocity and temperature.

**Figure 7:**
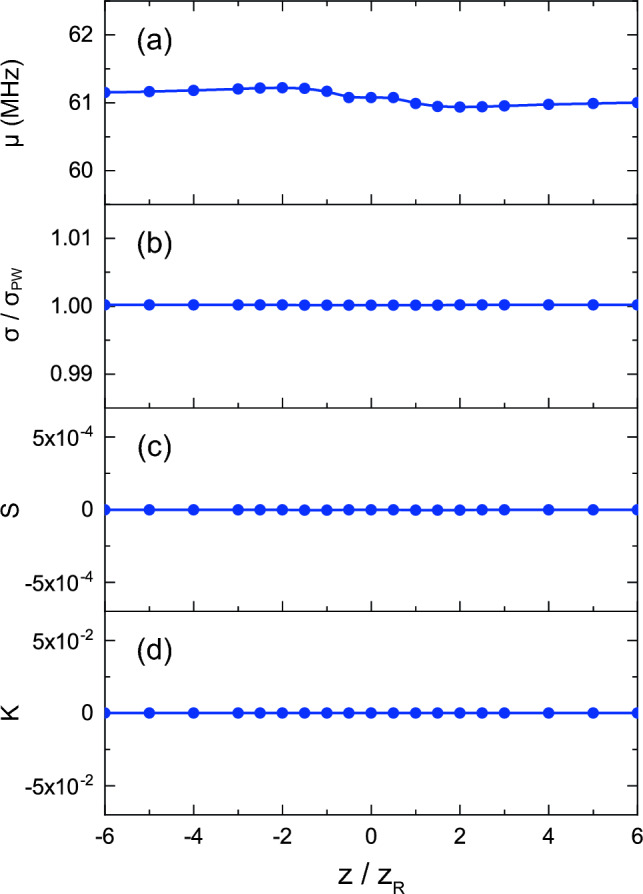
Axial dependence of each statistical quantity of aOVLIF spectra for $V_x=-3$ km/s, $T=0.1$ eV, $\ell =20$, $w_0=50$ $\mu$m, and $[\text {Im}(x_\text {s}), \text {Im}(y_\text {s})]=[-w_0/2, 0]$. The spectral width is normalized by that of the plane wave $\sigma _\text {PW}$.

**Figure 8:**
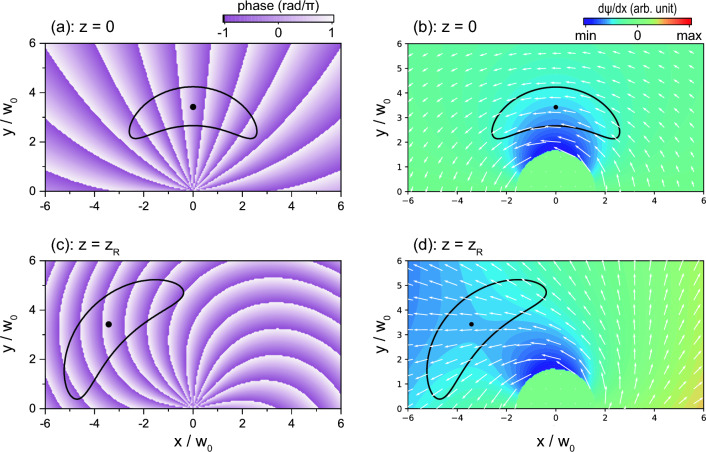
(**a**, **c**) Phase distribution of aOV beam for $\ell =20$ and $[\text {Im}(x_\text {s}), \text {Im}(y_\text {s})]=[-w_0/2, 0]$: (**a**) $z=0$ and (**c**) $z=z_\text {R}$. (**b**, **d**) Phase gradient in *x*-*y* plane, depicted by arrows: (**b**) $z=0$ and (**d**) $z=z_\text {R}$. The phase gradient distributions in the transverse flow direction ($\psi '_x= \partial \psi / \partial x$) are shown by the color contour maps, where the value in the central region ($\sqrt{x^2+y^2}< 1.8w_0$) is set to zero. The dots and solid black lines indicate the maximum intensity position and its 10% value at each axial position.

## Discussion

Here, we discuss how the rotation and expansion of the intensity distribution due to propagation depicted in Fig. 1c affect the LIF spectra. Figure 7 shows the axial dependence of the statistical quantities obtained from the aOVLIF spectra for $w_0=50$ $\upmu$m, $\ell =20$, $[\text {Im}(x_\text {s}), \text {Im}(y_\text {s})]=[-w_0/2, 0]$, and $V_x=-3$ km/s ($V_y=V_z=0$). The width of the spectrum in Fig. 7b is normalized by that for traditional LIF with a Gaussian beam ($\sigma _\text {PW}$). Note that the frequency shift and width are almost constant, and the skewness and kurtosis are almost zero. Although each statistic has a weak axial dependence, e.g., the odd distribution around $\mu (z=0)$ in Fig. 7a, the variation is not experimentally important. A frequency shift of 0.2 MHz corresponds to a transverse flow velocity of 12 m/s (0.4% of the assumed flow velocity) for the present beam parameters. However, the frequency resolution of the present calculations is not adequate to elaborate on this weak dependence on the frequency shift in the axial direction, and it will not be discussed further in this paper.

The fact that the characteristics of the spectrum change little in the propagation direction indicates that the phase structure changes with the propagation as well as the intensity distribution. Figure 8 shows phase distributions [(a) and (c)] and a contour map of the phase gradient in the transverse flow direction [(b) and (d)] perpendicular to $\varvec{k}$ at $z = 0$ and $z = z_\text {R}$, where the crescent-shaped solid lines are the contour lines at 10% of the maximum intensity on the beam cross-section (dots). In Figs. 8b,d, the phase gradient is also represented by arrows. As expected, it is confirmed that the phase structure convenient for the measurement of $V_x$ is maintained with propagation.

A simple analytical estimation can explain this property based on the axial dependence. In the present definition of the asymmetry parameter, the position of maximum intensity $(x^*, y^*)$ is placed at

9 $$\begin{aligned} x^*&= \frac{\hat{z} \text {Im}(x_\text {s}) + \text {Im}(y_\text {s})}{2} (1+g) , \ \ \sigma _\ell y^* = \frac{\hat{z} \text {Im}(y_\text {s}) - \text {Im}(x_\text {s})}{2} (1+g), \ \ g = \sqrt{1+ \frac{2|\ell |w_0^2}{D_\text {s}^2} }, \end{aligned}$$

and hence, the corresponding radius and azimuthal angle $(r^*, \varphi ^*)$ can be written as

10 $$\begin{aligned} r^*&= \frac{D_\text {s}(1+g)}{2} \sqrt{1+\hat{z}^2}, \ \ \varphi ^* = \tan ^{-1}(\sigma _\ell \hat{z}) + \varphi _0^*, \end{aligned}$$

where $\varphi ^*_0$ is a constant determined by the beam profile at $z=0$ [see Fig. 6a]. Here, Eqs. (9) and (10) are available for $D_\text {s} \ne 0$, and $\varphi ^* - \varphi ^*_0$ varies from $-\pi /2$ to $\pi /2$ in the range $-\infty \le \hat{z} \le \infty$. When an aOV beam satisfies the condition $|\ell | w_0^2 /D_\text {s}^2 \gg 1$, the asymmetry parameter in Eq. (10) vanishes and the radius can then be approximately expressed by $r^* \approx w(z) \sqrt{|\ell |/2}$, which is equivalent to the radius of the maximum intensity in an OV beam of TC $\ell$. As a result, the axial dependence of $(r^*, \varphi ^*)$ can be handled with that of a symmetric OV. It is clear that this approximation is valid in the case of $\ell \gg 1$ and $D_\text {s} \le O(w_0)$. Under these conditions, the additional Doppler shift due to the transverse flow at the position $(r^*, \varphi ^*)$ is given by $\delta (r^*, \varphi ^*)=(kr^*/R) V_r +(\ell /r^*) V_\varphi$ and we obtain

11 $$\begin{aligned} \delta (r^*, \varphi ^*)&= \frac{\sqrt{2|\ell |}}{w_0} \frac{(\hat{z} \cos \varphi ^* - \sigma _\ell \sin \varphi ^*)V_x+(\hat{z} \sin \varphi ^* + \sigma _\ell \cos \varphi ^*)V_y}{\sqrt{1+\hat{z}^2}}. \end{aligned}$$

By substituting Eq. (10) into Eq. (11), after a few steps, the additional Doppler shift can be rewritten as

12 $$\begin{aligned} \delta (r^*, \varphi ^*)&= -\sigma _\ell \frac{\sqrt{2 |\ell |}}{w_0} V_\perp \sin (\varphi _0^* - \varphi _\text {flow}), \end{aligned}$$

where the magnitude and angle regarding the transverse flow velocity stand for $V_\perp =(V_r^2+V_\varphi ^2 )^\frac{1}{2}=(V_x^2+V_y^2 )^\frac{1}{2}$ and $\varphi _\text {flow} = \tan ^{-1} (V_y / V_x)$, respectively. Equation (12) explains the sinusoidal dependence in Fig. 6, and it is also confirmed that the Doppler shift does not depend on the axial position. Moreover, the maximum frequency shift due to the transverse flow is obtained when $\varphi _0^*-\varphi _\text {flow}=\pm \pi /2$ indicating that the flow vector is parallel or antiparallel to the phase gradient.

When the intensity at the beam waist is adjusted as $\varphi _0^*=\pm \pi /2$, Eq. (12) is simplified as

13 $$\begin{aligned} \delta (r^*, \varphi ^*)_{\varphi _0^* = \pm \frac{\pi }{2}}&= \sigma _\ell \sigma _\text {s} \frac{\sqrt{2 |\ell |}}{w_0} V_x , \end{aligned}$$

where $\sigma _\text {s}$ is the sign of the imaginary part of the shift parameter ($\sigma _\text {s}=\text {sign}[\text {Im}(x_\text {s})$] in the present case). Note that in the present alignment of the aOV beam, Eq. (13) indicates that there is no sensitivity to $V_y$ because the phase gradient is oriented in the *x* direction. It is clear that Eq. (13) is identical to $Q^2=2|\ell |$. Although the previous result in Fig. 5 includes the effect of integration in the beam cross-section, it is interesting that the same relationship is obtained.

In a general case for a three-dimensional flow, the frequency shift of the LIF spectrum for an aOV beam of $[\text {Im}(x_\text {s}), \text {Im}(y_\text {s})]=[\pm w_0/2, 0]$ can be written as

14 $$\begin{aligned} \mu&\simeq \frac{1}{2\pi } \left( kV_z + \sigma _\ell \sigma _\text {s} \frac{\sqrt{2|\ell |}}{w_0}V_x \right) . \end{aligned}$$

This is an extended expression of the local Doppler-shifted absorption condition by Allen^20^ to the aOVLIF method. The sensitivity to transverse flow depends on $\sqrt{|\ell |}$, in contrast to Allen’s formula where it is proportional to $|\ell |$. Therefore, it is important to ensure sensitivity in aOVLIF by using a larger TC and a focused beam.

The fact that the shape of the LIF spectrum has no significant dependence on the beam propagation presents several advantages. Flow measurements using the aOVLIF method are tolerant to the alignment of the beam waist since the frequency shift is almost the same at any axial position. From another point of view, LIF measurements face a problem related to the excitation volume. The frequency shift of the LIF spectrum due to the transverse flow increases for smaller beams because the phase gradient perpendicular to the wavevector increases. Therefore, when the slower transverse flow has to be addressed, we need to use a focused beam after increasing the TC and the asymmetry parameter. However, since plasmas are frequently rarefied, the number of LIF target particles in a focused beam is not always sufficient; for example, the average distance between particles is 1 $\upmu$m even at a density of $10^{18}$ $\text {m}^{-3}$. When measuring rarefied plasmas, using an expanded beam far from the beam waist is helpful.

## Conclusions

We propose a new diagnostic method using aOV beams, aOVLIF, for plasma flow measurements, where the frequency shift of the LIF spectrum determines the transverse flow velocity. aOVLIF is also suitable for temperature evaluation. With a properly adjusted aOV beam, the frequency shift is proportional to both the transverse flow velocity and the square root of TC, which can be explained by the propagation property of the maximum intensity position. The axial dependence of spectra has also been examined, showing that the line-integration effect in the propagation direction does not affect the characteristics of the LIF spectrum.

The aOVLIF method works well for quantitatively determining the flow velocity across the beam by straightforwardly utilizing the spatial dependence of the resonance absorption condition. Moreover, the aOVLIF method can determine the three-dimensional velocity vector using a single optical path, which has not been achieved in previous LIF techniques. The aOVLIF method demonstrates that structured light enhances the versatility of laser spectroscopy in plasma experiments. The usefulness of the method will be confirmed experimentally in the near future.

## Methods

### OV beam production method

A tunable diode laser tuned at 448,379.1 GHz was used as a source beam to produce an OV beam. The Gaussian output beam was introduced into a spatial light modulator (SLM), which produces an OV beam as first-order diffraction light from a “fork” grating depicted by the liquid crystal display in the SLM. The hologram controls the TC, and the confirmed maximum TC in our system was $|\ell | = 50$. Details of the experimental setup have been previously described^27^.

When the Gaussian beam is aligned correctly with respect to the fork grating, a symmetric OV beam is produced. An aOV beam is generated by shifting the grating from the above condition^28^. In addition, the maximum intensity position of an aOV beam in the azimuthal direction ($\varphi ^*_0$ in Fig. 6) can also be controlled by this method. In the experiment, it was confirmed that the aOV beam is successfully generated in $D_\text {s} < w_0$.

### Visualization of phase structure of OV beams

The phase structure of an OV beam can be visualized by superimposing a Gaussian reference beam^29,30^. The two coaxially superimposed beams produce an interference pattern, as shown in Fig. 2. Since the wavefront of the Gaussian beam is almost flat in the cross-section, the brightness of the pattern reflects the phase structure of the OV beam. The phase change between adjacent bright points is $2\pi$ and, hence, the number of bright regions in the azimuthal direction gives the TC.

### Plasma parameters and LIF scheme

All the calculations were performed supposing a typical laboratory plasma for future proof-of-principle experiments. As the LIF target particles, we chose monovalent argon ions with a temperature of 0.1 eV and a typical transverse flow velocity of a few km/s. These parameters are relevant in our experiments^12,13,31^. We adopt a frequently utilized LIF scheme: the argon ions in the lower energy state $3d \, ^4F_{7/2}$ are excited to the upper state $4p\, ^4D^0_{5/2}$ by absorbing laser photons of 448,379.1 GHz^32^. Throughout this paper, we also assume that the LIF intensity is proportional to both the laser intensity and the population in a lower energy level, i.e., a linear regime, where the excitation and de-excitation processes occur between the two energy levels. The latter assumption is consistent with ignoring the Stark and Zeeman effects.

### Velocity distribution function and corresponding LIF spectrum

For a plasma in thermal equilibrium, the velocity distribution function (VDF) for the ions is assumed as to be an isotropic Maxwell–Boltzmann distribution with a drift velocity $\varvec{V} =(V_x,V_y,V_z )$,

15 $$\begin{aligned} f(\varvec{\upsilon })&= \left( \frac{1}{2\pi V_\text {t}^2} \right) ^\frac{3}{2} \exp \left[ - \frac{(\upsilon _x-V_x)^2+(\upsilon _y-V_y)^2+(\upsilon _z-V_z)^2}{2V_\text {t}^2}\right] , \end{aligned}$$

where $V_\text {t}=\sqrt{k_\text {B}T/M}$ is the thermal velocity, *T* is the temperature, *M* is the ion mass, and $k_\text {B}$ is the Boltzmann constant. By substituting the resonant absorption condition $\delta =\nabla \psi \cdot \varvec{\upsilon }$ into Eq. (15), we obtain an expression of the VDF in frequency space, $g(\delta )$, which satisfies the relation $\int _{-\infty }^\infty g(\delta ) \text {d}\delta = \int _\text {all} f(\varvec{\upsilon }) \text {d}\varvec{\upsilon }$.

The Doppler shift in angular frequency for non-relativistic particles with velocity $\varvec{\upsilon }$ is represented by $\delta _\text {D}= \omega - \omega _0 = -\nabla \psi \cdot \varvec{\upsilon }$, where $e^{\text {i}\psi }$ gives the spatial phase distribution of the beam and $\omega _0$ is the Doppler frequency for stationary particles in the reference frame. In the LIF method based on the resonance absorption process, the resonance absorption condition with a Doppler shift can be expressed as $\delta = -\delta _\text {D}=\nabla \psi \cdot \varvec{\upsilon }$. Setting the wavenumber in the *z* direction ($\varvec{k} = k\varvec{e}_z$) and considering the resonant absorption condition at $z = 0$, we can confirm the expressions for the Doppler shift $\delta =k\upsilon _z$ for a plane wave and $\delta \approx k\upsilon _z+ \ell \upsilon _\varphi /r$ for a symmetric OV beam.

Since we have assumed that the number of fluorescence photons is proportional to the population of ions satisfying the resonant absorption condition and beam intensity, the local LIF intensity is given by $|u|^2 g$ as a function of $\delta$. In actual circumstances, LIF measurements have been carried out in a finite volume determined by the beam size and the observation region of the collecting optics. By assuming that the accumulation of contributions from each position in the beam cross-section determines the LIF intensity, the LIF spectrum at each axial position along the beam path can be expressed as

16 $$\begin{aligned} I_\text {LIF}(\delta , z)&\propto \left[ \frac{1}{2\pi V_\text {t}^2 ({\psi '_x}^2+{\psi '_y}^2+k^2)} \right] ^\frac{1}{2} \int _\text {all} |u|^2 \exp \left[ -\frac{(\delta + {\psi '_x}V_x + {\psi '_y} V_y + kV_z)^2 }{2V_\text {t}^2 ({\psi '_x}^2+{\psi '_y}^2+k^2)}\right] \text {d}S, \end{aligned}$$

where $\psi '_j=\partial \psi / \partial x_j$ ($j=1, 2, 3$) stands for each component of the phase gradient. Details of the derivation of LIF spectra have been previously described^22^. In traditional LIF ($\psi '_x=\psi '_y=0$), the LIF spectrum in Eq. (16) satisfies $S=K=0$. Here, the phase gradient in the *z* direction was approximated by $\psi '_z \approx k+O[|\ell |/(k^2 w_0^2 )]\approx k$ [see Eq. (17)], and the vertical phase gradients $\psi '_x$ and $\psi '_y$ were numerically calculated.

### Approximation of axial phase gradient

The axial phase gradient for a Laguerre–Gaussian beam is given by

17 $$\begin{aligned} \frac{\partial \psi }{\partial z} =\psi '_z&= k + \frac{kr^2}{2(z^2+z_\text {R}^2)} \left( 1 - \frac{2z^2}{z^2+z_\text {R}^2} \right) -\frac{(2p+|\ell |+1)z_\text {R}}{z^2+z_\text {R}^2}. \end{aligned}$$

At $z=0$, the second term becomes unity, and the third term is $2|\ell |/(kw_0^2 )$. For $k \gg 1$, $p=0$, and $|\ell | \gg 1$, the phase gradient is $\psi '_z\approx k[1-2|\ell |/(kw_0 )^2 ]$. For an OV beam with $\nu = 448,379.1$ GHz, $w_0=50$ $\mu$m, and $|\ell |=100$, the approximation $\psi '_z \approx k$ is valid because $2|\ell | / (kw_0 )^2 \sim 10^{-3} \ll 1$. The axial component of the phase gradient is almost independent of the asymmetry parameter.

### Numerical setup

The spatial resolution of the calculation was $w_0/20$ and the integration was carried out within a radius of $40w_0$ from the beam center. In the LIF spectrum calculation, the frequency resolution was set to 1 MHz when an accurate evaluation was required and 20 MHz otherwise.

### Evaluation of statistical quantities of LIF spectrum

Numerically obtained LIF spectra are discrete, consisting of *N* total data number. Using the normalized LIF spectrum $I_\text {LIF} (\nu _j )$ satisfying $(1/N) \sum _{j=1}^N I_\text {LIF}(\nu _j)=1$, the statistical quantities for the discrete spectra are given by

18 $$\begin{aligned} \mu&= \frac{1}{N} \sum _{j=1}^N \nu _j I_\text {LIF} (\nu _j), \ \ \sigma ^2 = \frac{1}{N} \sum _{j=1}^N (\nu _j - \mu )^2 I_\text {LIF}(\nu _j), \nonumber \\ S&= \frac{1}{\sigma ^3 N} \sum _{j=1}^N (\nu _j - \mu )^3 I_\text {LIF}(\nu _j), \ \ K =-3+ \frac{1}{\sigma ^4 N} \sum _{j=1}^N (\nu _j - \mu )^4 I_\text {LIF}(\nu _j). \end{aligned}$$

The discrete laser frequency $\nu _j$ is detuned by the resonant absorption frequency for stationary ions.

## Data Availability

The datasets used and analysed during the current study available from the corresponding author on reasonable request.
